# A comprehensive review on promising anti-viral therapeutic candidates identified against main protease from SARS-CoV-2 through various computational methods

**DOI:** 10.1186/s43141-020-00085-z

**Published:** 2020-11-03

**Authors:** Ekampreet Singh, Rameez Jabeer Khan, Rajat Kumar Jha, Gizachew Muluneh Amera, Monika Jain, Rashmi Prabha Singh, Jayaraman Muthukumaran, Amit Kumar Singh

**Affiliations:** 1 Department of Biotechnology, School of Engineering and Technology, Sharda University, Greater Noida, U.P P.C. 201310 India; 2 Department of Biotechnology, IILM College of Engineering & Technology, Greater Noida, U.P India

**Keywords:** 3CLpro, In silico, Inhibitors, Structure-based virtual screening (SBVS), Ligand-based virtual screening (LBVS), Drug-repurposing

## Abstract

**Background:**

The COVID-19 pandemic caused by SARS-CoV-2 has shown an exponential trend of infected people across the planet. Crediting its virulent nature, it becomes imperative to identify potential therapeutic agents against the deadly virus. The 3-chymotrypsin-like protease (3CLpro) is a cysteine protease which causes the proteolysis of the replicase polyproteins to generate functional proteins, which is a crucial step for viral replication and infection. Computational methods have been applied in recent studies to identify promising inhibitors against 3CLpro to inhibit the viral activity.

**Main body of the abstract:**

This review provides an overview of promising drug/lead candidates identified so far against 3CLpro through various in silico approaches such as structure-based virtual screening (SBVS), ligand-based virtual screening (LBVS) and drug-repurposing/drug-reprofiling/drug-retasking. Further, the drugs have been classified according to their chemical structures or biological activity into flavonoids, peptides, terpenes, quinolines, nucleoside and nucleotide analogues, protease inhibitors, phenalene and antibiotic derivatives. These are then individually discussed based on the various structural parameters namely estimated free energy of binding (*ΔG*), key interacting residues, types of intermolecular interactions and structural stability of 3CLpro-ligand complexes obtained from the results of molecular dynamics (MD) simulations.

**Conclusion:**

The review provides comprehensive information of potential inhibitors identified through several computational methods thus far against 3CLpro from SARS-CoV-2 and provides a better understanding of their interaction patterns and dynamic states of free and ligand-bound 3CLpro structures.

## Background

Coronaviruses are enveloped, positive-sense, single-stranded RNA viruses that can cause respiratory, enteric, hepatic and neurologic diseases in mammals and birds [1, 2]. They belong to the family coronaviridae, subfamily coronavirinae, and are classified into four genera, namely alpha, beta, gamma and delta [3]. Most members of this family are enzootic with only a few species that infect humans (alpha and beta coronaviruses) [4]. The following known coronaviruses, Human coronavirus NL63 (HCoV-NL63), Human coronavirus 229E (HCoV-229E), Human coronavirus OC43 (HCoV-OC43), Human coronavirus (HCoV-HKU1), Severe acute respiratory syndrome coronavirus (SARS-CoV), SARS-CoV-2 and Middle East respiratory syndrome coronavirus (MERS-CoV), are known to infect humans [4]. Out of the seven human infecting coronaviruses, some remain relatively harmless (229E, NL63, OC43, HKU1) causing respiratory tract infections while others such as SARS-CoV and MERS-CoV are known etiological agents for two epidemics in 2002 and 2012, respectively causing more than 10,000 cumulative cases [3]. The recent pandemic associated with COVID-19 caused by SARS-CoV-2 has shown an unprecedented trend in the number of cases with over 37 million infected and more than one million mortalities worldwide [5].

Coronaviruses possess the largest known viral genome ranging from 26 to 32 kb with unique replication machinery consisting of proteins to protect the viral genome and sequester it from the host’s immune system [2]. The viral genome features a *5*′ cap and *3*′ poly (A) tail that allows it to act as an mRNA for translation. The *5*′ end contains a leader sequence and an untranslated region (UTR) followed by the replicase gene encoding within two Open Reading Frames (ORFs), the non-structural proteins (nsps) which occupy two-thirds of the genome [6]. The *3*′ end of the genome contains the structural proteins, i.e. spike (S), membrane (M), envelope (E) and nucleocapsid (N), and the accessory proteins are interspersed throughout the genome [7]. The S glycoprotein mediates the entry of the virus into the host cell via binding of its receptor binding domain (RBD) to the angiotensin-converting enzyme 2 (ACE2), a membrane protein expressed in various organs mainly lungs, kidney and intestines [4, 8, 9]. The cleavage of S glycoprotein by membrane protease such as TMPRSS2 or by endosomal protease like cathepsin L is critical for viral entry into the host cell [9–11]. The E proteins are essential for virus assembly and formation of ion channels in the viral membrane [12, 13]. The N protein and viral RNA form the ribonucleoprotein and the M protein aids in viral assembly and stabilization of the N-RNA complex and helps in structural determination of the envelope [13–15].

The viral genome possesses ORFs that encode the replicase polyproteins that code for the non-structural proteins (nsps). The production of replicase proteins begins with the translation of two ORFs 1a and 1ab through a 1-ribosomal frameshift mechanism, which produces two polyproteins pp1a and pp1ab [16]. This is followed by proteolytic processing of polyproteins by two virally encoded cysteine proteases, papain-like protease (PLpro, nsp3) and 3C- like protease (3CLpro, nsp5) or Main protease (MPro), which are essential for the maturation of 16 nsps [17, 18]. Evidence suggests that these nsps form the replication-transcription complex, which is localized in endoplasmic reticulum-derived membrane forming a microenvironment for protection of the viral RNA [19]. These nsps include various proteins that perform a plethora of crucial activities essential for viral survival. The nsp12, an RNA-dependent RNA polymerase, is responsible for the synthesis of viral RNA and utilizes nsp7 and 8 as cofactors for enhanced functioning [20–22]. The viral helicase (nsp13) is critical for replication since it possesses the ability to unwind double stranded DNA or RNA [23]. Several nsps such as nsp14 (N7-MTase) and nsp16 (2′-OMTase) are responsible for RNA modification and require nsp10 as a cofactor [24–26]. The nsp15, a uridylate-specific endoribonuclease, is responsible for sequestering the viral genome from the host immune system [27]. Together, these nsps perform critical functions crucial for viral replication, expression and vitality.

The current pandemic situation has caused the scientific community to search for effective therapeutic methods against the virus in a time-sensitive fashion. One such approach popularly applied for the identification of potential therapeutic agents is through computational approaches. Various drug-like or lead-like candidates have been identified or repurposed against various drug target proteins of SARS-CoV-2 through a structure-based virtual screening, targeting these proteins essential for viral replication. One such target with indispensable activity towards replication and infection is 3CLpro which proteolytically cleaves 11 sites on pp1a and pp1ab for the production of replicase proteins, i.e. nsps [28]. This prerequisite property of 3CLpro for viral replication makes it an ideal and unique drug target for antiviral therapy.

**Fig. 1:**
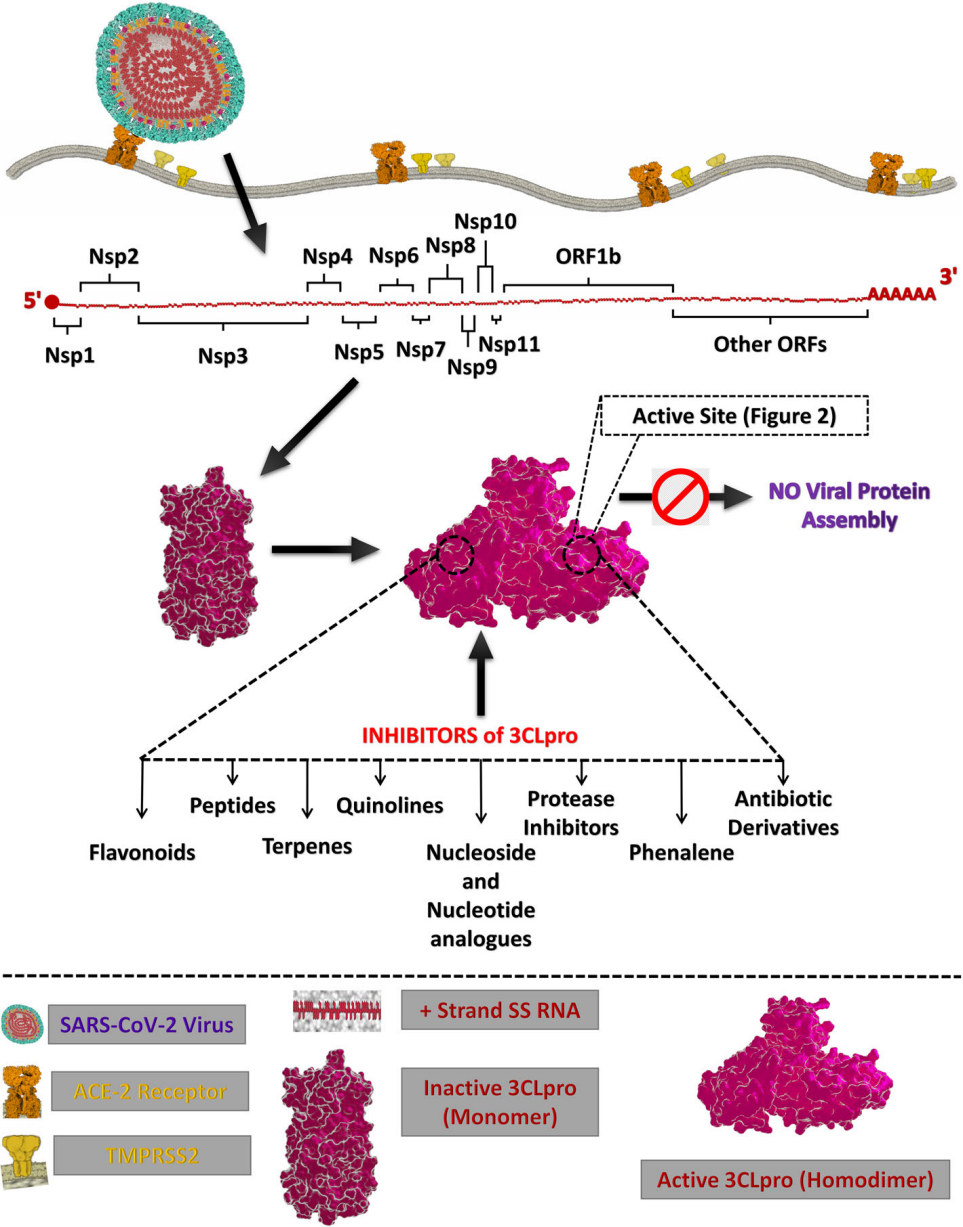
Schematic representation for the localization and function of possible inhibitor classes against 3CLpro from SARS-CoV-2

This perspective dives into the inhibitors that have been identified against 3CLpro of SARS-CoV-2 through in silico approach in recent studies (Fig. 1). This review aims at providing a cumulative source of computationally identified promising drug-like or lead-like candidates against 3CLpro from SARS-CoV-2 which provides a better insight into the various computational drug discovery techniques such as structure-based virtual screening, ligand-based virtual screening, pharmacophore-based virtual screening, drug repurposing, molecular docking, quantitative structure activity relationship (QSAR), quantitative structure property relationship (QSPR) and molecular dynamics simulations.

## Main text

### 3-Chymotrypsin-like protease (3CLpro)

The maturation of coronaviruses involves an intricate proceeding of robust proteolytic processing of the replicase polyproteins to regulate viral gene expression and replication. The majority of cleavage events associated with the polyproteins are performed by the 3CLpro which begins with autolytic cleavage of itself followed by cleavage of 11 interdomain sites to generate functional proteins like a helicase, an exoribonuclease, an RNA-dependent RNA polymerase, an endoribonuclease and a 2′-OMTase [28, 29]. Several crystal studies elucidate the 33.8 kDa 3CLpro as a homodimer with the two protomers aligned at right angles to each other [30]. Each protomer comprises 306 amino acid residues and consists of three functional domains characteristic of 3CLpro. The domain I (residues 8–101) and II (residues 102–184) are antiparallel *β*-barrels representative of cysteine proteases of the chymotrypsin family [31]. The domain III contains an arrangement of five *α* helices into an antiparallel globular cluster connected to domain II via an extended loop region (residues: 185–200) [30, 31]. The substrate-binding site is the deep cleft between domain I and II lined by hydrophobic residues with the catalytic site present in the centre of the cleft [32].

### Active site and mechanism

**Fig. 2:**
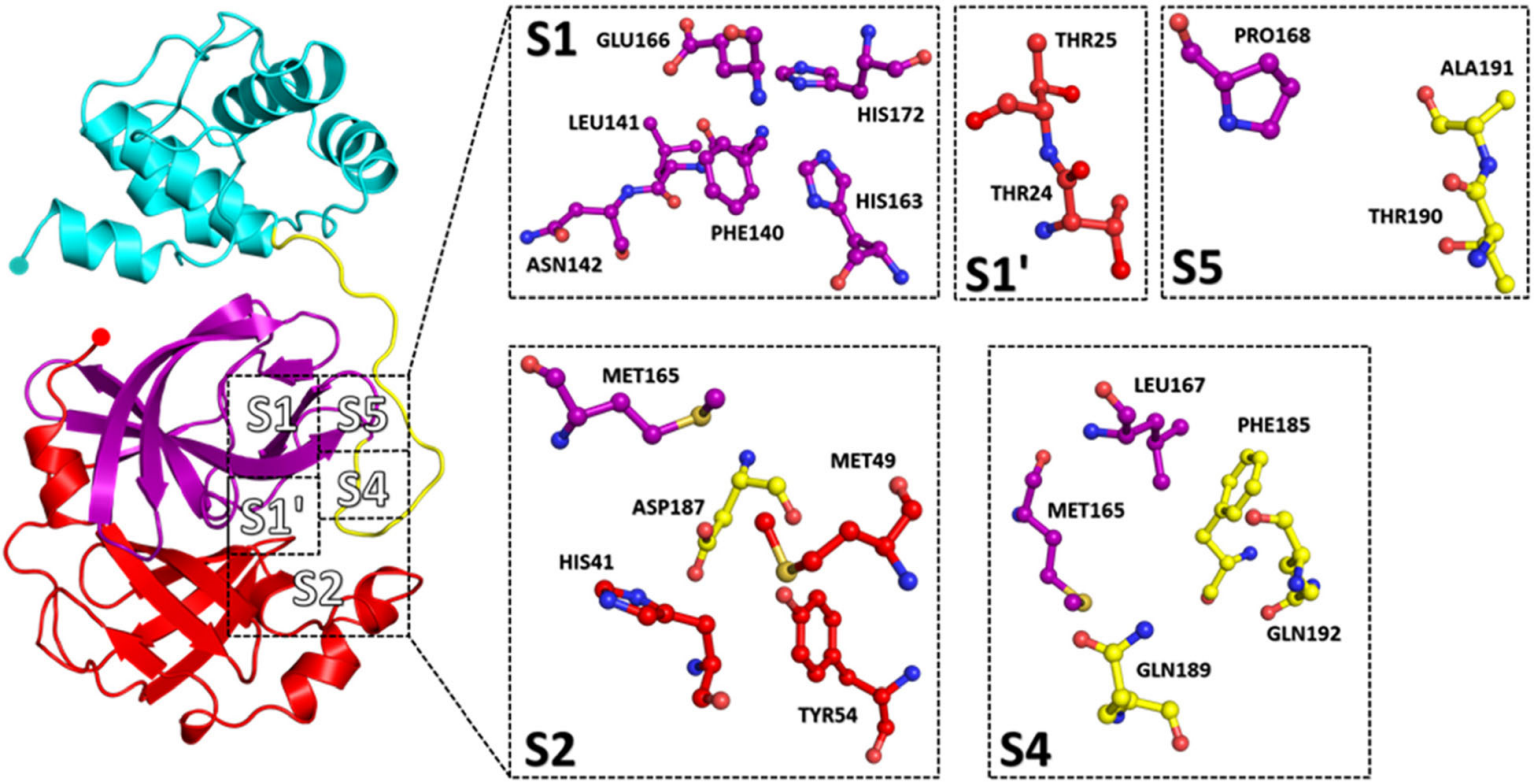
Substrate binding subsites (S1, S2, S4, S5, S1′) of 3CLpro from SARS CoV-2 (Domain I—red, domain II—purple, domain III—cyan, extended loop—yellow)

3CLpro features a unique Cys-His dyad (Cys145 and His41) as its catalytic residues and is surrounded by other residues which confer substrate specificity. The conservation of specificities of the substrate, i.e. *N*-terminal peptide of 3CLpro prefers Thr-Ser-Ala-Val-Leu-Gln as positions P6 to P1, which interact with the substrate-binding site residues [33–35]. In the S1 subsite, the imidazole side chain of conserved histidine residue interacts with the carboxamide side-chain of P1; this reaction is generally accepted to be conducive of specificity for glutamine residue at P1 [6, 32]. Strong hydrogen bonds between Gln-P1 and His163, Gln-P1 and Phe140 ensure suitable binding of the substrate into the S1 site [6, 36]. The side chain of Leu-P2 and Thr-P4 is stabilized by deep hydrophobic S2 and S4 subsite, respectively. Ser-P5 and Thr-P6 residues interact with Pro168 and Ala191 residues of the enzyme through van der Waals interactions [34, 35, 37]. On the *C* terminal side of the substrate, the P1’ position is occupied by a small residue like Ala, Ser and Gly residues which directly interact with the S1’ shallow subsite through van der Waals interactions [33]. A long side-chain leucine is accommodated by the hydrophilic S2’ subsite of the protease [33]. These structural attributes primarily of the S1, S2 and S4 subsite (Fig. 2) of the protease are essential for substrate binding and have been exploited for drug designing and discovery against SARS-CoV-2 due to their crucial role in the activity of 3CLpro.

The catalytic mechanism of 3CLpro initiates with the deprotonation of thiol group of Cys145 residue followed by a nucleophilic attack of resulting Sulphur anion on the substrate carbonyl carbon [16, 38]. This step results in the release of a peptide product with an amide terminus and in concert His41 residue is restored to its deprotonated form [16, 17]. The penultimate step is the hydrolysis of the resultant thioester to release a carboxylic acid which then leads to the regeneration of the protease [16, 17, 39]. This process of hydrolysis of the substrate by a general acid-base mechanism defines the catalytic action of 3CLpro.

### Inhibition of 3CLpro

A recent study dives into the inhibition of 3CLpro from SARS CoV-2 with the help of a computationally designed Michael acceptor inhibitor N3 (PubChem CID: 146025593) which has been known to show inhibitory activity towards several 3CLpro including those from SARS and MERS. Through molecular docking studies, it was found that N3 oriented in a suitable pose within the substrate-binding site [31]. The efficacy of this inhibitor was analysed with enzymatic assays which inferred that N3 is a potent time-dependent inhibitor of 3CLpro. The same study includes a structural elucidation of the 3CLpro with N3 complex at 2.1 Å, which expounds the interactions between the inhibitor and crystal structure of 3CLpro (PDB ID: 6 LU7). The *Sγ* atom of Cys145 residue forms a covalent bond with the *Cβ* of the vinyl group of N3 confirming the addition of the Michael inhibitor. The lactam at P1 forms a hydrogen bond with His163 residue in the S1 subsite. At P2, the side chain of Leu residue inserts into the hydrophobic S2 subsite whereas the side chain of Val residue at P3 is solvent exposed indicating its ability to tolerate a variety of functional groups. Ala residue at P4 interacts with a small hydrophobic pocket while P5 and bulky benzene at S1′ makes van der Waals interactions with residues. It is also evident that N3 makes multiple hydrogen bonds with the residues of the substrate binding pocket, which helps to lock the inhibitor into the pocket [31, 35].

Another study elucidates the interactions of the inhibitor carmofur with 3CLpro from SARS-CoV-2, which shows a similar trend where the fatty acid moiety of carmofur is linked to the *Sγ* atom of Cys145 residue through a covalent bond [40]. In both studies, it is evident that the inhibitors cause a covalent modification of the catalytic Cys145 residue of SARS-CoV-2’s 3CLpro though it is interesting to notice that the mechanism of covalent modification can be different. It is also of interest to notice that through superposition analysis of the structures of 3CLpro, it is observed that domain III and the surface loops appear to be the most variable regions [31]. However, the substrate-binding pockets present in the cleft between domain II and domain III are highly conserved which makes them an ideal and unique drug target for the designing of potential antiviral compounds that have potent inhibitory activity against the virus [31, 41].

### Potential inhibitors of 3CLpro from SARS-CoV-2

Various compounds identified through in silico assisted virtual screening and drug repurposing, i.e. in silico approaches, have been delineated as potential inhibitors of the catalytic site of 3CLpro. In silico approach provides researchers with a method to design promising drug or lead candidates or repurpose the existing drugs that show inhibitory activity based on the rationale of structure-based drug designing which targets the essential structural features of a protein and identifies the potent inhibitors based on the concept of estimated free energy of binding and the formation of various intermolecular interactions such as hydrogen bonds, hydrophobic interactions and van der Waals interactions. Several computational studies have been conducted to identify inhibitors against 3CLpro from SARS-CoV-2 through virtual screening of a library of synthetic and natural compounds (Table 1).

**Table 1:** Existing promising and potential molecules against 3CLpro from SARS CoV-2

S/No	PubChem CID	Binding free energy (kcal/mol)	Tools used	Interacting residues	Refs
1	5,7,3′,4′-Tetrahydroxy-2′-(3,3-dimethylallyl) isoflavone(11610052)	− 29.57	Molecular operating environment (MOE)	Thr25, Thr26, Leu26, His41, Val42, Ser46, Met49, Tyr54, Leu141, Asn142, Gly143, Ser144, Cys145, His163, His164, Met165, Glu166, Leu167, Pro168, Asp187, Arg188, Gln189, Thr190, Ala191, Gln192	[42]
2	Cyanidin 3-glucoside(197081)	− 8.4	Autodock Vina	Glu166, Asn142, His163, Gln189, Asp187, Thr26, Met49, Gly143, His164, Gln189, Cys145	[43]
3	Baicalin(64982)	− 8.1	Autodock Vina	Gly143, Leu141, Gln189, Met165, Asn142, Glu166, Pro168	[43]
4	Glabridin(124052)	− 8.1	Autodock Vina	Leu141, Glu166, Met49, His41, Met165	[43]
5	Quercetin 3-vicianoside(44259139)	− 8.3	Autodock Vina	His163, Glu166, Ser144, Leu114, Gly143, Thr26, Arg188, Asp187, Met165, His164, Gln189, His41, Thr25, Asn142, Phe140, Cys145	[44]
6	Myricitrin(5281673)	− 8.9	Autodock Vina	Tyr54, Asp187, Arg188, Leu141, Phe140, His172, Glu166, Ser144, His164, His163, Leu37, Asn142, Gly143, Thr26, Cys145, Met165, Met49, His41	[45]
7	Oolonghomobisflavan-A(14520989)	− 75.5	CDOCKER	Thr25, Asn142, His163, Glu166, Arg188, His164, Gly143, Met165, His41, Phe140, Leu141, His172, Ser144, Thr24, Cys145, Leu27, Met49, Asp187, Gln189, Gln192, Val186, Thr190, Pro168, Leu167	[46]
8	Birinapant(49836020)	− 8.1	Glide Schrodinger Suite	Thr25, Thr26, Leu26, His41, Val42, Ser46, Met49, Tyr54, Leu141, Asn142, Gly143, Ser144, Cys145, His163, His164, Met165, Glu166, Leu167, Pro168, Asp187, Arg188, Gln189, Thr190, Ala191, Gln192	[47]
9	Carfilzomib(11556711)	− 8.6	Glide Schrodinger Suite	Cys145, His164, Glu166, Gln192, Asn142, Gln189, His41	[48]
10	6-Oxoisoiguesterin(21575473)	− 9.1	Autodock Vina	Arg189, Met49, Met165, Cys145	[49]
11	22-Hydroxyhopan-3-one(21582894)	− 8.6	Autodock Vina	Arg189, Met49, Met165, Cys145	[49]
12	Crocin(5281233)	− 8.2	Autodock 1.5.4 tools	Thr135, Asn133, Thr199, Lys137, Lys5, Phe3, Arg4, Arg131, Asp197	[50]
13	Carnosol(442009)	− 8.2	Autodock 1.5.6 tools	Cys145, His164, Glu166, Gln189, Met165, Arg188, Cys44, Met49, His41, Thr45, Thr25, Thr26, Leu27, Gly143, Ser46, Asn142	[51]
14	Rosmanol(13966122)	− 7.9	Autodock 1.5.6 tools	Cys145, Gly143, Thr25, His41, Met49, Asn142, His143, Glu166, Leu141, Ser144, Met165, Phe140	[51]
15	Withanone(21679027)	− 4.4	Glide Schrodinger Suite	Thr24, Thr25, Thr26, Leu27, His41, Met49, Tyr54, Asn142, Gly143, His164, Met165, Glu166, Arg188, Asp188, Gln189, Cys145	[52]
16	Nelfinavir(64143)	− 7	Autodock Vina	His163, Gln189	[53]
17	CMPD23B	− 118.6	Igemdock	Arg40, Tyr54, Cys85, Phe181, Arg188, Arg40, Tyr54, Glu55, Met82, Asn84	[54]
18	Amodiaquine(2165)	− 7.4	Glide Schrodinger Suite	Leu141, ARG187, Glu166, His41	[55]
19	Remdesivir(121304016)	− 7.9	Glide Schrodinger Suite	Gln191, Ala192, Thr190, Gln189, Arg188, Asp187, Leu141, Asn142, Gly143, Ser144, Cys145, His164, Met165, Glu166, Leu167, Pro168, Thr26, Leu27, Met49, Tyr54, His41	[56]
20	Ribavirin(37542)	2	Glide Schrodinger Suite	Leu4, Gln189, Met49, Val3, Ser46, Cys145, Thr25, Thr24, Thr45	[57]
21	Telbivudine(159269)	2	Glide Schrodinger Suite	Thr190, Leu50, Glu47, Ser46, Met49, Val3, Gln189	[57]
22	Lopinavir(92727)	− 10.8	MMPBSA.py module of AMBER16	Met49, Met165, Pro168, Gln189, His41, Ala46, Met49, Glu166, Leu167, Leu187, Gln189, Ala191, Ala193	[58]
23	Ritonavir(392622)	− 14.9	MMPBSA.py module of AMBER16	Leu27, His41, Met49, Phe140, Asn142, Gly143, His164, Met165, Glu166, Asn142, Gly143, Ser144, Cys145, Met165, Glu166, Asp187, Gln189	[58]
24	Saquinavir(441243)	− 9.8	Glide Schrodinger Suite	His41, Cys44, Tyr54, Met49, His172, Glu166, Met165, His164, His163, Phe140, Leu141, Asn142, Gly143, Ser144, Cys145, Thr25, Thr26, Leu27, Asp187, Arg188, Gln189, Thr190, Ala191	[56]
25	Simeprevir(24873435)	− 10	Autodock Vina	Glu166, Met165, Gln189, Phe140, Cys145, Asn142, Leu27, His41, Ser46	[59]
26	Paritaprevir(45110509)	− 9.8	PyRx v0.8(Autodock Vina)	Ser46, Met49, Leu50, Cys145, Pro168, Thr24, Thr25, Leu27, His41, Cys44, Thr45, Glu47, Phe140, Gly143, Glu166, Gln189	[60]
27	Hypericin(3663)	− 10.7	CDOCKER	Gly143, Glu166, Asn142, Glu166, His41, Cys145, Asn142, Glu166, Gln189, Met165	[43]
28	Eravacycline(54726192)	− 8.8	Glide Schrodinger Suite	Met49, Gln189, Tyr190, Gln192, Glu166, Met165, Asn142	[48]
29	Valrubicin(454216)	− 9.2	Glide Schrodinger Suite	His41, His164, Asn142, Met165, Glu166, Gln189	[48]
30	Viomycin(135398671)	− 77.2	CDOCKER	Glu166, Phe140, Asn142, His164, Ser144, Gly143, His41, Met49, Met145, Gln189, Arg188, Asp187, His163, Cys145	[61]
31	Elbasvir(71661251)	− 9.9	Glide Schrodinger Suite	Thr25, Thr26, His41, Met49, Met165, Gln189, Thr190.	[48]
32	Fluvastatin(446155)	− 7.7	Autodock Vina	Glu166, His163.	[62]
33	Methisazone(667492)	− 6.8	Glide Schrodinger Suite	His41, His49, Tyr54, Cys145, Met165, Gln166, Leu167, Pro168, Asp187, Arg188, Gln189, Thr190, Gln192.	[63]

#### Flavonoids

These are a group of polyphenolic plant metabolites with a general structure consisting of two phenyl rings and a heterocyclic ring [64]. Various libraries of such phytochemicals have been computationally screened against 3CLpro, and promising molecules have been identified [42–44, 46]. One such molecule, 5,7,3′,4′-Tetrahydroxy-2′-(3,3-dimethylallyl) isoflavone, displayed a binding affinity of −29.57 kcal/mol when docked using molecular operating environment (MOE) with 3CLpro (PDB Id: 6 LU7) through hydrogen bonding residues namely His41, Phe140, Gly143 and Glu166 and hydrophobic interactions with other key substrate residues [42]. Another study identified Cyanidin 3-glucoside as an inhibitor with an estimated binding free energy of −8.40 kcal/mol using Autodock Vina [65]; it forms six hydrogen bonds and three hydrophobic interactions, one of which is with the catalytic Cys145 residue [43]. In the same study, Baicalin interacts with 3CLpro through one hydrophobic interaction, six hydrogen bonds, one pi-sulphur interaction with the catalytic Cys145 residue, whereas Glabridin forms one electrostatic and five hydrophobic interactions. Interestingly, both Baicalin and Glabridin show a similar estimated binding free energy of − 8.10 kcal/mol. Another flavonoid Quercetin 3-vicianoside interacts with His163, Glu166, Ser144, Leu114, Gly143 and Thr26 residues through hydrogen bonding and shows hydrophobic interactions with ten key residues and showed binding free energy of − 8.30 kcal/mol [44]. Myricitrin, an anti-tussive molecule, through ADME analysis showed high solubility and bioavailability and displayed an estimated binding free energy of − 8.9 kcal/mol through Autodock Vina [65]. The catalytic residues such as His41 and Cys145 interacted with a pi-alkyl and pi-sulphur bond with the aromatic scaffold of the ligand. It shows six hydrogen bonds with additional van der Waals interactions that stabilize the interaction between protein and ligand [45]. In a drug repurposing study, Oolonghomobisflavan-A molecule identified from tea plant showed minimum binding free energy from molecular mechanics Poisson–Boltzmann surface area (MM-PBSA) and a CDOCKER [66] interaction energy of 75.54 kcal/mol. It was observed to manifest seven hydrogen and several other stabilizing van der Waals interactions. The evaluation of MD simulations results showed that it was able to show strong interaction and higher stability with the binding pocket of 3CLpro and also had the potential to affect the dimerization of the protease [46]. The results of such molecular docking studies have delineated flavonoids as potent inhibitors against 3CL Pro from SARS-CoV-2.

#### Peptides

Several drug molecules have been identified against 3CLpro which sports the chemical structure typical of a peptide classifying them as peptide inhibitors. Birinapant is one such drug, a peptidomimetic small molecule which displays a docking score of − 8.14 using VS Workflow of Glide Schrodinger Suite and MM-GBSA binding free energy of −105.15 kcal/mol and forms a hydrogen bond with catalytic Cys145 residue [47]. MD simulation analysis performed using Desmond v3.6 module [67] highlighted that Birinapant granted maximum stability to the protein through hydrogen bonds appearing at hot spot residues in the proceeding of the simulation. Carfilzomib is a tetrapeptide epoxyketone and an approved anticancer drug. It has also been repurposed in computational studies as an inhibitor for 3CLpro showing a docking score of − 8.6 using Glide flexible docking [68] and an MM-PBSA-WSAS binding free energy of −13.8 kcal/mol, and in conjugation, the MD trajectories from RMSD (root-mean-square deviation) fluctuation analysis show overall stability [48].

#### Terpenes

Terpenes constitute a large hydrocarbon class constructed from five-carbon isoprene units which are combined in a great variety of skeletons [69]. 6-Oxoisoiguesterin, a bisnorterpenes, displayed a binding affinity of −9.1 kcal/mol using Autodock Vina 4.2 [65] when docked against 3CLpro (PDB ID: 6 LU7) of SARS-CoV-2 [49]. It formed a conventional hydrogen bond with Arg189 residue and created an alkyl and pi-alkyl stacking with Met49, Met165 and Cys145 residues. Another terpenoid 22-Hydroxyhopan-3-one had a binding affinity of − 8.6 kcal/mol with one hydrogen bond to Lys137 residue and alkyl, pi-alkyl interaction with Leu287, Leu286, Leu286 and Tyr239 residues [49]. The ADME parameters of both the drugs indicate them to be non-carcinogenic, low toxicity and aqueous solubility of < 0, which shows them to be promising inhibitors. A library of therapeutic compounds from Moroccan medicinal plants was computationally investigated against 3CLpro (PDB ID: 6 LU7) which identified Crocin (binding free energy − 8.20 kcal/mol), a carotenoid pigment of saffron forming eight hydrogen bonds, two alkyl attractions, two carbon-hydrogen bonds, and attractive charge interactions [50]. Another study of chemical compounds from Indian spices analysed two diterpenes Carnosol (*ΔG* − 8.20 kcal/mol) and Rosmanol (*ΔG* − 7.99 kcal/mol) showing stable estimated binding affinity (Raccoon and MGLTools-1.5.6 software by Autodock [70]) and ADME properties. When docked against 3CLpro (PDB ID: 6Y84), both drugs showed interactions with the active site residues, and MD simulation (Desmond package by Schrodinger [67]) of 50 ns inferred strong hydrogen bonding interactions and stronger stability of the protein-ligand complex [51]. Another study found Withanone, an active withanolide of *W. somnifera*, displayed active binding to the substrate pocket of 3CLpro and showed binding free energy of −34.51 ± 9.63 kcal/mol from MM/GBSA (molecular mechanics/generalized born surface area) and a Glide flexible docking score of −4.42. Forming one hydrogen bond with the catalytic Cys145 residue, several hydrophobic interactions, pi-pi stacking with several key residues and in the dynamic state through MD simulations using Desmond [67]with OPLS3e force field from Schrodinger it showed nine hydrogen bonds [71] (including one with His41 residue) and several other stabilizing interactions which assist to its efficacy as a promising inhibitor [52]. These studies indicate a variety of chemical compounds derived from natural sources that inhibit the activity of the viral protease in a virtual setting aiding the supposition of these compounds as promising inhibitors.

#### Quinolines

These compounds have a characteristic structure of a benzene ring fused with a pyridine making it a double-ringed heterocyclic aromatic organic compound [72]. Nelfinavir, an antiretroviral drug, is an isoquinoline that has been studied profoundly for its activity against 3CLpro from SARS-CoV-2. In one in silico study, it shows an estimated binding affinity of −7.0 kcal/mol using Autodock Vina [65]. Through two independent MD simulations (IBM Power-cluster), it is observed that nelfinavir occupies almost the whole catalytic pocket of 3CLpro and is stabilized through internal hydrogen bonds, two hydrogen bonds with His163, Gln189 residues and significant hydrophobic interactions [53]. A library of noscapine and its derivatives was screened against 3CLpro (PDB ID: 6 LU7) in which CMPD23B was identified as a potent virtual inhibitor showing efficient binding free energy and stabilizing interactions [54]. Another study identified Amodiaquine, an antimalarial aminoquinoline showing a docking score of − 7.42 kcal/mol (Glide Module), formed four hydrogen bonds with Leu141, Arg187 residues and salt bridge interaction with Glu166 residue and a pi-pi stacking with catalytic His41 residue [55].

#### Nucleoside and nucleotide analogues

A popularly identified drug molecule against 3CLpro from SARS-CoV-2 through in silico methods is Remdesivir, an adenosine triphosphate analogue with potential antiviral activity against several RNA viruses. In one study, it showed a binding score of − 7.9 using Covdock-Schrodinger suite (covalent docking module) [73] and MM/GBSA binding free energy of −65.19 kcal/mol, forming a covalent, irreversible bond with the catalytic Cys145 residue and three hydrogen bonds with several other stabilizing interactions. An MD simulation of 50 ns using GROMACS 2018.1 showed a significant decrease in the RMSD value of 3CLpro, RMSF (root-mean-square fluctuation) analysis shows reducing the flexibility of protein backbone, Rg (radius of gyration) values were in agreement with the previous results, and PCA (principal component analysis) inferred the higher stability of drug bound protein [56]. Another virtual screening study using Schrodinger glide docking module [68] identified two nucleoside analogues Ribavirin, an antiviral for hepatitis C, and Telbivudine, an antiviral for hepatitis B, as likely inhibitors of 3CLpro [57].

#### Protease inhibitor

In various recent computational endeavours, protease inhibitors of different viruses such as HIV, MHV, hepatitis C and SARS-CoV, have been virtually screened against 3CL protease of SARS-CoV-2. Undoubtedly, two drugs, namely lopinavir and ritonavir, have been studied because of their past clinical relevance with SARS and MERS. Both are HIV-1 protease inhibitors with known antiviral activity and have hence served as potential drugs in various studies and even been used as control drugs in other studies [74–76]. In one specific study, the binding free energy prediction by MM/PBSA using MMPBSA.py module of AMBER16 [77] for lopinavir and ritonavir was found to be −10.89 and − 14.93 kcal/mol, respectively. MM/GBSA using MMGBSA.py module of AMBER16 [77] gave a value of − 13.83 kcal/mol for lopinavir and − 27.28 kcal/mol for ritonavir in binding free energy calculations. Through residue binding free energy calculation, key residues were identified for binding of the drugs to the catalytic site of 3CLpro. Four residues (Met49, Met165, Pro168 and Gln189) and nine residues (Leu27, His41, Met49, Phe140, Asn142, Gly143, His164, Met165 and Glu166) were important for the binding of lopinavir and ritonavir, respectively. MD simulations in the studies are conducive of robust binding between the ligands and the binding site of 3CLpro, indicating them as promising drugs against SARS-CoV-2 [58]. In another study, Saquinavir, an antiretroviral protease inhibitor of HIV/AIDS, was found to have a docking score of − 9.85 using Covdock by Schrodinger suite and displayed binding energy of − 72.17 kcal/mol through MM/GBSA calculations. It was found to make a covalent bond with catalytic Cys145 residue and five hydrogen bonds with binding pocket residues of 3CLpro. In MD simulations (using GROMACS 2018.1) of the protein-drug complex, it was found that there was a significant decrease in the RMSD value (0.039 Å) of 3CLpro when bound with the drug and also shows that binding of Saquinavir led to diminished fluctuations in the protein backbone. Throughout the simulation, Saquinavir showed average hydrogen bonding of 2, and through PCA, it was observed that it reduced the trace of covariance value to a lower degree of functional motions [56]. Moreover, this was additionally supported that this specific drug molecule increases the stability of 3CLpro. Another study identified Simeprevir with estimated binding free energy value (using Auto Dock Vina) of − 10.0 kcal/mol with three hydrogen bonds (Glu166, Gly143 and Asn142 residues) [59]. A drug repurposing study identified Paritaprevir, a protease inhibitor of HCV, with an estimated binding free energy of − 9.8 kcal/mol using PyRx v0.8 [78]. Paritaprevir formed one hydrogen bond with Ser46 residue and four pi interactions (including one with catalytic Cys145 residue) and van der Waal interactions. The MD simulation of the protein-drug complex performed using GROMACS 2019 implicated that Paritaprevir maintained the structural stability and integrity of 3CLpro, the catalytic dyad remains highly stable, and a maximum of six hydrogen bonds was observed in the proceeding of the simulation. PCA inferred that protein was more flexible during its affiliation with the drug; this along with other parameters delineates Paritaprevir as a promising inhibitor against 3CLpro [60].

#### Phenalene

Hypericin is an antidepressant with a potent antiviral activity that sports a structural scaffold of phenalene. It shows a binding affinity of − 10.7 kcal/mol (CDOCKER [79]) and manifests a pi-alkyl interaction with catalytic residue Cys145, four hydrogen bonds and five hydrophobic interactions with 3CLpro structure (PDB ID: 6 LU7). MD simulations performed using GROMACS version 5.1 observed induced local flexibility of key active residues when hypericin is bound to the protein. Other MD parameters corroborate the prediction of significant inhibition of 3CLpro by hypericin [43].

#### Antibiotic derivatives

Another series of drugs for inhibition of 3CLpro is antibiotic derivatives which have been repurposed in several studies. Eravacycline is a synthetic halogenated tetracycline antibiotic [80], and valrubicin is a chemotherapeutic drug which is a derivative of anthracycline antibiotic (doxorubicin) [81]. In a study, Eravacycline had a docking score of − 8.8 while valrubicin showed a docking score of − 9.2 when Glide flexible docking [68] was performed. The MD simulations of both the protein-drug complexes showed stable interactions [48]. Another study identified Viomycin, a tuberactinomycin antibiotic, formed eight hydrogen bonds with 3CLpro (PDB ID: 6 LU7) during docking studies with a CDocker energy of 77.29 kcal/mol. Allosteric binding of the drug was studied by a 100 ns MD simulation which indicated low RMSD values aiding to the stability of the protein-ligand complex. Interaction studies show Viomycin forming eight H-bonds with key substrate binding site residues and a residue responsible for dimerization (Phe140) [61]. These derivatives, along with others, represent a strong class of promising inhibitors for 3CLPro against SARS-C0V-2.

#### Imidazole

Compounds containing a five-carbon aromatic ring with nitrogen atoms at 1,3 positions are classified as imidazoles [82]. Elbasvir is a known direct acting antiviral used for the treatment of hepatitis C virus (HCV) [83]. A recent docking study of elbasvir displayed a glide score of − 9.9 with significant interactions with Thr25, Thr26, His41, Met49, Met165, Gln189 and Thr190 residues. The MM-GBSA binding free energy decomposition was performed to identify hot spots which included the catalytic His41 and Gln189 residues. An RMSD analysis of Elbasvir-3CLpro complex showed a stable structural pattern throughout the MD simulation [48].

#### Indoles

Compounds classified as indoles are characterized with a benzene ring fused with pyrrole ring [84]. In a recent study, Fluvastatin displayed a binding affinity of − 7.7 kcal/mol with significant interactions with Glu166 and His163 residues. Another similar study explored the binding score of Methisazone with 3CLpro (PDB Id: 5R80) through Glide flexible docking, which displayed a score of −6.8. The hydroxyl group of the ligand formed H-bond with Glu166 residue, another H-bond, was formed between Thr190 and -NH_2_ of ligand. The indole moiety of Methisazone displayed pi-pi stacking with His41 residue [63].

## Conclusion

The high transmission rate of SARS-CoV-2 has led to the current COVID-19 pandemic with infections in over 188 countries/regions and over a million fatalities worldwide. This virulent attribute of the virus has led to fastidious research endeavours to find effective and potent inhibitors of the virus. While clinical trials for an ideal vaccine continue, it is crucial to identify alternative antiviral candidates to keep the virus from spreading any further. As traditional drug discovery is a long and laborious task, a repurposing approach has been applied by various researchers to identify potential inhibitors of SARS-CoV-2. Computer aided drug discovery has been robustly used to identify potential inhibitors to molecular targets of SARS-CoV-2. Such computational methods are excellent priming steps in drug discovery as they help propose a promising target and in conjugation with molecular dynamics simulations and more recently with artificial intelligence, the effectiveness of drugs can be predicted. It is important to note that in vivo studies in conjugation with in silico analysis are essential to understand the biological relevance of a promising drug candidate. Computational approaches effectively reduce the screening time and help in lead identification.

Out of the various targets of SARS-CoV-2, the 3CLpro is evidently one of the most investigated viral protein. The 3CLpro is responsible for proteolysis of the viral replicase polyproteins that produce the proteins essential for the viral life cycle. This indispensable activity makes it an attractive target for inhibition studies. Virtual screening studies of various molecules have shown high levels of binding affinities, and these results have often been corroborated with molecular dynamics studies. The compounds reviewed in the study showed appreciable screening results and interacted with key residues that are essential for the activity of 3CLpro. Interaction studies of the compounds show that the ligands effectively interacted with the catalytic subsites of 3CLpro including critical residues like His41, Cys145, Gln189, Glu166, Asn142 and Met 49 residues. These results help present data that can be applied to further extensive experimental analysis that can aid in the identification or discovery of a lead agent for inhibition of 3CLpro.

The present study extensively analyses research literature to report a comprehensive insight into the computational studies that are being carried out presently to identify potential candidates against 3CLpro. Moreover, the present study focused on the comprehensive collection of potential and promising drug or lead candidates against 3CLpro from SARS-CoV-2, which are identified by using various in silico techniques. Our on-going and future works are directed towards to collect the experimental details of promising molecules reported so far against 3CLpro from SARS-Cov-2. As the research regarding SARS-CoV-2 continues to grow in an unprecedented fashion, this review study aims to present critical data coherently to create an inclusive report of current research studies.

## Data Availability

All data generated or analysed during this study are included in this manuscript.

## Abbreviations

SARS-CoV-2: Severe acute respiratory syndrome 2
COVID-19: Coronavirus disease
3CLpro: 3-Chymotrypsin like protease
Mpro: Main protease
MD: Molecular dynamics simulations
MM-PBSA: Molecular mechanics Poisson–Boltzmann surface area
MM/GBSA: Molecular mechanics generalized born surface area
RMSD: Root-mean-square deviation
RMSF: Root-mean-square fluctuation
Rg: Radius of gyration
PCA: Principal component analysis
